# Formulation development, characterization, and evaluation of sorafenib-loaded PLGA–chitosan nanoparticles

**DOI:** 10.3389/fphar.2024.1465363

**Published:** 2024-10-09

**Authors:** Abdul Mateen, Abad Khan, Ismail Khan, Lateef Ahmad, Amjad Khan, Abdul Salam

**Affiliations:** ^1^ Department of Pharmacy, University of Swabi, Swabi, Pakistan; ^2^ HBS Institute of Healthcare & Allied Health Sciences, Islamabad, Pakistan; ^3^ Department of Pharmacy Kohat University of Science and Technology (KUST) Kohat, Kohat, Pakistan; ^4^ Institute of Pathology and Diagnostics Medicine, Khyber Medical University, Peshawar, Pakistan

**Keywords:** drug delivery, sorafenib, polymer, PLGA, biodegradable, cytotoxicity

## Abstract

The basic purpose of this work was to develop environmentally friendly, biodegradable, and biocompatible polymeric nanoparticles of sorafenib that can effectively release the desired drug in a customized and controlled manner for targeting hepatocellular carcinoma. The solvent evaporation technique was employed for the synthesis of sorafenib-loaded PLGA–chitosan nanoparticles, followed by various experimental specifications and compatibility studies using poloxamer 407 as the stabilizer. The best nanoparticles thus synthesized were selected to be used for cytotoxicity investigations through *in vitro* and *in vivo* assessments. For the *in vitro* drug release tests, the dialysis bag diffusion technique was used. For both chitosan nanoparticles and PLGA loaded with sorafenib, a biphasic release pattern was found, exhibiting a protracted release lasting 10 days after a 24-h burst release. As experimental animals, rabbits were utilized to evaluate different *in vivo* pharmacokinetic properties of the selected formulations. Plasma samples were extracted with acetonitrile and analyzed through the developed HPLC method. Pharmacokinetic parameters such as AUC_0-t_, C_max_ MRT, Vd, and half-life (t_1/2_) were enhanced significantly (*p* ≤ 0.001), while clearance was considerably decreased (*p* ≤ 0.001) for the chosen synthesized nanoparticles in contrast to the commercially accessible sorafenib formulation (Nexavar^®^). The cytotoxicity of the reference drug and sorafenib-loaded PLGA and chitosan nanoparticles was calculated by performing an MTT assay against HepG2 cell lines. The developed polymeric sorafenib nanoformulations possess the appropriate physicochemical properties, better targeting, surface morphology, and prolonged release kinetics. The pharmacokinetic parameters were improved significantly when the results were compared with commercially available sorafenib formulations.

## 1 Introduction

In Europe and United States, hepatocellular carcinoma is known to be a leading cause of liver malignancy, making it a major cause of cancer-related mortality, and the obvious reason for this is poor lifestyle and unhealthy eating habits (Dimitroulis et al., 2017). Due to the high ratio of prevalence, hepatocellular carcinoma occupies a leading position in the world. It is responsible for taking almost half a million human lives worldwide and is considered the third most common cause of death (Michielsen et al., 2005). The diagnosis and treatment of this fatal disease have been a challenge so far, but strategies include orthotopic liver transplant, hepatic resection, ablative therapies, and chemotherapy with available cytotoxic drugs (Dimitroulis et al., 2017). In the early stages of hepatocellular carcinoma, various treatment techniques like surgical resection, local ablation, and liver transplant can effectively enhance the chances of survival of patients. Once the carcinoma has reached an advanced stage, no effective treatment is available to ensure the life expectancy of the patient (Lin et al., 2012). The major reported cause of the development of HCC to an advanced stage is the decreased expression of carcinoma-suppressing genes and enhanced expression of oncogenes, resulting in the successive development of HCC, which ultimately causes the sequential hyperactivation of an important enzyme known as Raf/MEK/extracellular signal-regulated kinase (ERK) (Avila et al., 2006).

Currently, a major limiting factor in diagnosing and treating this fatal disease is the highly toxic profile of the anti-cancer drugs along with poor sensitivity and specificity. To resolve these important issues, a novel technique has been in use experimentally for the past few years and in real patients, known as nanotechnology (nanoparticle synthesis), for both diagnosis and treatment of cancer. This nanoparticle-based treatment is quite effective because it has drastically reduced the toxic effects of chemotherapeutic drugs. In order to target the cancerous tissue or tumor specifically, both active and passive approaches are now utilized(Surendiran et al., 2009). One of the most extensively used anti-cancer drugs, sorafenib, is marketed by Bayer (Whippany, NJ, USA). Under the brand name Nexavar^®^, this drug has been approved by the FDA for the treatment of advanced renal cell carcinoma and hepatocellular carcinoma. Receptor tyrosine kinases and Raf serine/threonine kinases are both actively engaged in tumor cell production and tumor development and are inhibited by oral sorafenib, a tyrosine kinase receptor inhibitor. Sorafenib is mostly prescribed to patients for whom surgery cannot be performed, and hence, this drug is effectively used against such carcinomas (Iyer et al., 2010).

The most important consideration while formulating nanoparticles is their ability to effectively release the bound drug in a customized and controlled manner along with another important parameter, the compatibility of the nanoparticles with the physiology of the human body (Fonseca et al., 2002). In this regard, a very effective polymer known as poly-lactic-co-glycolic acid (PLGA), which has excellent properties of biocompatibility, biodegradability, and neutrality to the immune system, has been approved by the European Medicines Agency, the FDA, and other regulatory agencies. This polymer is readily hydrolyzed in the body to produce glycolic acid and a lactic acid biocompatible monomer, which makes it acceptable to be widely used in the formulation of nano-particles as they are both metabolized by the body’s citric acid cycle. In contrast, chitosan (CS) is a semi-processed natural cationic linear polysaccharide composed of 1–4 glucosamine units linked to N-acetyl-glucosamine (Jalali et al., 2016). Chitosan has unique properties of being bio-degradable, bio-compatible, bio-active, non-toxic, and non-immunogenic and is used for its ability to penetrate the molecular surface of the mucosa (Jalali et al., 2011; Li et al., 2018).

Another polymer, poloxamer 407, is a combination of three blocks of a co-polymer with polyethylene oxide (hydrophilic portion), which is cationic, along with a polypropylene oxide portion (hydrophobic portion). “The nanoparticle surface is covered by the hydrophobic end of poloxamer 407, whereas the section that loves water involves creating a hydrophilic layer in the direction of the aqueous medium” (Redhead et al., 2001). The bio-adhesive nature and enhanced drug solubility make this surfactant amphiphilic in nature. “The FDA has approved poloxamer 407 as a bio-active component for use in injectable, topical, ophthalmic, and other drug preparations” (Dumortier et al., 2006).

The purpose of this study was to formulate PLGA nanoparticles loaded with sorafenib for the efficient treatment of hepatocellular carcinoma. Important parameters to be achieved by this formulation include physicochemical characterization, pharmacokinetic evaluation, *in vitro* cytotoxicity, and drug release experiments. Following a thorough investigation, the suggested formulations for the treatment of hepatocellular carcinoma have been determined to be both safe and effective. The benefit of the created nanoformulations is that they make use of polymeric stabilizers, which can increase stability, bioavailability, and solubility.

## 2 Materials and methods

### 2.1 Materials

Sorafenib (purity ≥99.9%) was received from Qi Lu Laboratories, Republic of China. Low molecular weight chitosan and poloxamer 407 were obtained from Sigma-Aldrich, Germany. PLGA (75:25, Resomer^®^ RG 756 H, MW 76000–115000 Da) was obtained from Evonik, Germany. Acetonitrile (purity ≥99.9%), Dialysis tubing-Dia 27/32–21.5mm, 30 M MWCO ∼ 12,000–14,000 Da was obtained from Sigma-Aldrich, Germany. Distilled water ultra-pure was used to prepare the desired solution.

### 2.2 Pre-formulation analysis

#### 2.2.1 Sample preparation

In order to study the interactions between sorafenib, the polymers, and the stabilizers, the physical mixtures containing these moieties (1:1 w/w) were investigated. The samples were maintained at 40°C ± 2˚C and 75° ± 5% RH for 30 days (Maddiboyina et al., 2023).

#### 2.2.2 Compatibility studies

These mixtures were stored for a period of 30 days, and the interactions were investigated at 40°C ± 2°C and 75% ± 5% RH (Maddiboyina et al., 2023). At each sample point, substance concentration, physical state, and FTIR spectra were checked for any potential drug excipient incompatibilities.

#### 2.2.3 Drug content analysis by UV-vis spectrophotometer

Samples containing excipients, the drug, and the polymer kept under stress were generated, and the amount of drug content was determined by analysis. After being dissolved in ACN solution, samples, and standard solutions were examined and compared. Drug content was measured in triplicate at the moment of sampling, and the results are displayed as mean +SD.

#### 2.2.4 Analysis by Fourier-transform infrared spectroscopy (FTIR)

To check for any incompatibility in the sample, FTIR was used. The potassium bromide pellet technique was employed for sample formation. A 1% amount of the sample was combined with already dried potassium bromide and then ground for at least 5 min. The obtained sample was then further crushed to a level where it could be easily compacted by pressing with reasonable force. The samples were analyzed in the region of 400–4,000 per centimeter (Gautam et al., 2022).

#### 2.2.5 Evaluation of the physical consistency of samples

Visual inspection was conducted on the samples for any type of variation in texture and color. Changes in physical consistency were used to monitor the physical and chemical interactions between the excipients, medication, and polymer.

### 2.3 Preparation of nanoformulations

Sorafenib-loaded CS-modified PLGA nanoparticles were made by the emulsion–solvent–evaporation technique (Table 3). PLGA and sorafenib were dissolved in ethyl acetate, which formed the organic phase. The organic phase was mixed slowly and steadily using a dropper with the water-containing medium containing chitosan (0.2%, w/v) dissolved in 1% (v/v) of the acetic acid solution containing poloxamer. After overnight stirring, the solvent was evaporated, and chitosan-modified nanoparticles were obtained (Yang et al., 2009). The unbound drug was separated by centrifugation (12,000 r/min, 15 min). The sorafenib-loaded nanoparticles were then lyophilized for 48 h.

### 2.4 Physicochemical characterization

#### 2.4.1 Particle size and polydispersity index (PDI)

The compositions were analyzed in order to measure the size and PDI by dynamic light scattering (DLS at 90^o^C and 25^o^C) using Zeta sizer (ZS-90, Malvern Instruments and Malvern, UK). Distilled water was used for diluting the nanoformulations where needed. Malvern software was used to obtain the average of the three reported results and perform a statistical analysis.

#### 2.4.2 Zeta potential

The surface charge was measured with Zetasizer. Each sample was measured three times using the zeta potential principle, which is the electrophoretic mobility in an electric field.

#### 2.4.3 Drug loading and encapsulation ability

Centrifugation (15,000 rpm, 25°C, and 30 min) was used to determine the drug loading (DL) and encapsulation efficiency (EE) (%, w/w) of sorafenib in the developed formulations. After thorough centrifugation, the absorbance of the precipitated nanoparticles was measured at 235 nm by UV spectroscopy. The following formulae were used to calculate the percentage of loading and encapsulation (Ghaferi et al., 2020):

$$Encapsulation\;Effeciency=\frac{Weight\;of\;Drug\;in\;Nanoparticles}{Weight\;of\;Drug\;initially}\;X\;100,$$

$$Percent\;Drug\;Loading=\frac{Weight\;of\;Drug\;in\;Nanoparticles\;}{Weight\;of\;Nanoparticles\;}\;X\;100.$$

#### 2.4.4 Scanning electron microscopy

The above method evaluated the morphological appearance of the particles. The morphology of the substance was studied using SEM. To make the sample conductive, it was prepared using standard SEM techniques. The sample was then inspected for structural integrity.

#### 2.4.5 X-ray diffraction study

The design of X-ray diffraction analysis of crushed sorafenib, polymers, various excipients, and nanoformulations was tested for amorphous, crystalline, and semi-crystalline properties. At 3°–80° (2θ), the design was adopted and used.

### 2.5 *In vitro* analysis

#### 2.5.1 Drug release studies

Sorafenib formulations' *in vitro* release experiments were carried out utilizing the dialysis diffusion technique. The membrane, which has a molecular mass between 12,000 and 14,000 Da, was sliced so that it could hold 2 mL of re-dispersed nanoformulations and was made watertight or closed up at both ends. Then, in a shaking water bath at 37°C and 60 rpm, the specific membrane was dialyzed against 100 mL of PBS (pH 7.4). At time intervals of 0.5, 1, 2, 4, 6, 8, 12, 24, 36, 48, 72, 96, 120, 144, 168, 192, 216, and 240 h, a sample (2 mL) was taken out and its drug release was examined. For each sample, the same volume of the dialyzing medium was replaced. Using a UV spectrophotometer, the drug content of each sample was measured at 265 nm. Each sample was analyzed in triplicate (Wan et al., 2021). Several release kinetic models were employed to check the drug release processes (Türkeş and Açıkel, 2024).

#### 2.5.2 In vitro Cytotoxicity

Sorafenib’s *in vitro* cytotoxicity was established by carrying out an MTT (yellow tetrazolium salt, 3-(4, 5-dimethylthiazol-2-yl)-2,5- diphenyl tetrazolium bromide) assay using HepG2 cell lines. They represent a good model to test the cytotoxicity of compounds for liver cancer. Cells were kept in DMEM with 10% FBS and 1% antibiotics (100 U/mL penicillin). They were then seeded in a 96-well plate at a density of 1.0 × 10^4 cells per well and incubated for 24 h at 37°C in a 5% CO_2_ atmosphere. The medium was discarded, and the cells were treated with varying concentrations of the test compounds: 1 μg/mL, 2.5 μg/mL, 5 μg/mL, 10 μg/mL, 20 μg/mL, and 40 μg/mL (Sakhi et al., 2022). Then, 48 h after incubation (Shah et al., 2023), 20 μL of the MTT solution (5 mg/mL) was pipetted into each well and incubated for another 4 h. The medium was subsequently discarded, and the formazan precipitate was dissolved in DMSO. Using the microplate reader, the absorbance of the mixtures was measured at 570 nm in triplicate. Cytotoxicity was expressed as follows (Sakhi et al., 2022; Avula et al., 2023):

$$Percent\;Viability=\frac{Absorbance\;of\;sample}{Absorbance\;of\;control}\;X\;100.$$

### 2.6 *In vivo* study

#### 2.6.1 Pharmacokinetic studies

New Zealand rabbits weighing between 1.5 and 2.0 kg were purchased from a breeding facility of the Department of Pharmacy, University of Peshawar, KP, Pakistan, for conducting *in vivo* pharmacokinetic studies. The Ethical Committee of the Department of Pharmacy, University of Swabi, approved the study design by letter no. Pharm/EC/035. Water and food were made available to the rabbits. To obtain better results, the study animals were housed in a stress-free setting. The dosage was injected into the marginal ear vein of the rabbits at a rate of 10 mg/kg mass. For the purpose of the sorafenib test and reference formulations, the subject animals were split into two groups. At certain periods of time (0.25, 0.5, 1, 2, 4, 6, 8, 12, 24, 36, 48, 72, 96, 120, 144, 168, 192, 216, and 240 h), samples of blood were drawn into heparin tubes. The samples collected were then centrifuged at 8,000 rpm for at least 10 min at 4°C. Samples were collected and kept in Eppendorf tubes at −20°C and analyzed by HPLC.

Different pharmacokinetic parameters like elimination half-life (t_1/2_), peak plasma concentration (C_max_), the area under the plasma concentration-versus-time curve (AUC0-∞), clearance (cL), the area under the curve (AUC), the volume of distribution (V_d_), and mean residence time (MRT) were measured with the help of PK-Summit software.

### 2.7 Statistical analysis

The mean (X), % RSD, and standard deviation (SD) were utilized to quantify sorafenib in the collected samples. Student’s t-test was used to compare the treatment groups at *p* < 0.05.

## 3 Results

### 3.1 Pre-formulation analysis

#### 3.1.1 Drug excipient compatibility study

Research was conducted on the compatibility of drug excipients, and the FTIR spectra obtained are shown in Table 1.

**TABLE 1:** Compatibility studies.

Time	Test	Specimen 01	Specimen 02	Specimen 03	Specimen 04	Specimen 05	Specimen 06	Specimen 07	Specimen 08	Specimen 09
Day 01	FTIR spectra	Compliant	Compliant	Compliant	Compliant	Compliant	Compliant	Compliant	Compliant	Compliant
	Physical consistency	Compliant	Same as above	Same as above	Same as above	Same as above	Same as above	Same as above	Same as above	Same as above
Day 30	FTIR spectra	Compliant	Same as above	Same as above	Same as above	Same as above	Same as above	Same as above	Same as above	Same as above
	Physical consistency	Compliant	Same as above	Same as above	Same as above	Same as above	Same as above	Same as above	Same as above	Same as above

#### 3.1.2 Physical consistency of samples

Specimens were made using medication and excipient paired mixes (1:1), kept under stress for a period of 1 month, and subsequently, visually examined for color and texture changes. The physical consistency of the samples, as seen in Table 2, did not change.

**TABLE 2:** Drug content analysis.

Drug content (%)						
Time	Characteristic	Standard drug	Samples			
			09	10	11	12
Day 01	Drug content	99.87	99.45	98.41	99.76	99.63
Day 30		99.89	98.91	99.12	99.41	99.84
Day 60		99.66	99.01	99.21	98.94	99.04
Day 90		99.81	99.21	99.02	99.11	98.85

#### 3.1.3 Determination of drug content

The drug concentrations of standard drugs and samples were determined, and the findings are shown in Table 2.

#### 3.1.4 FTIR spectroscopy

Chemical incompatibility between the drugs and excipients was investigated by FTIR spectroscopy. Specimens were kept for a period of 30 days at 40°C ± 2°C and 75% ± 5% RH, and FTIR studies were carried out.

**FIGURE 1:**
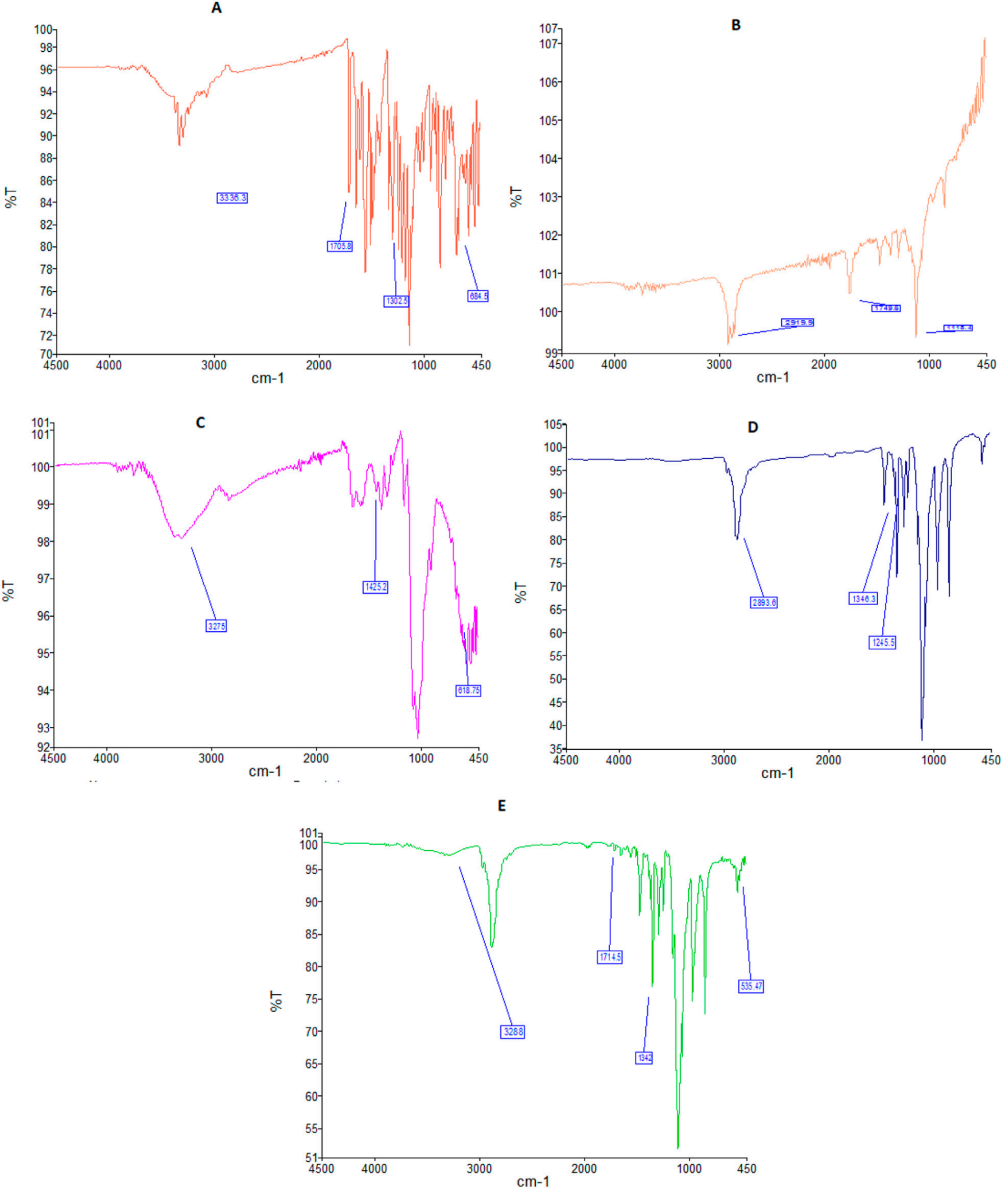
FTIR spectra of sorafenib **(A)**, PLGA **(B)**, chitosan **(C)**, poloxamer **(D)**, and nanoformulation **(E)**.

A band at 684 cm^-1^ in the FTIR spectra of free sorafenib (Figure 1A) is linked to the C–Cl stretching of the drug. The C=O of the carbonyl group was at 1,302 cm^-1^, the C=O of the amide group was at 1,705 cm^-1^, and the N–H stretching of the amide group was at 3,336 cm^-1^.

Figure 1B shows the FTIR spectrum of PLGA, having a peak at 1,118 cm^−1^, which is characteristic of the C–O group. A band at 1,749 cm^−1^ is attributed to the existence of the C=O of the carbonyl group. A band at 2,919 cm^−1^ is attributed to the C–H straightening out.

Figure 1C shows the FTIR band of chitosan at 618 cm^-1^, which is attributed to the C=N extending vibration. The FTIR spectrum of CS at 1,425 cm^-1^ is attributed to the CO–NH_2_ and NH_2_ kinds of chitosan. The broad band at 3,275 cm^−1^ corresponds to the extending vibration of chitosan with combined bands of the NH_2_ and OH groups.

The FTIR spectrum of the poloxamer is shown in Figure 1D. The bands at 1,245 and 1,346 cm^−1^ belong to the C–O–C group, whereas the peak at 2,893 cm^−1^ reflects the O–H stretching.

FTIR spectra of sorafenib nanoformulation are shown in Figure 1E, with bands at 535, 1,342, 1,714, and 3,288 cm^−1^.

#### 3.1.5 X-ray diffraction studies

**FIGURE 2:**
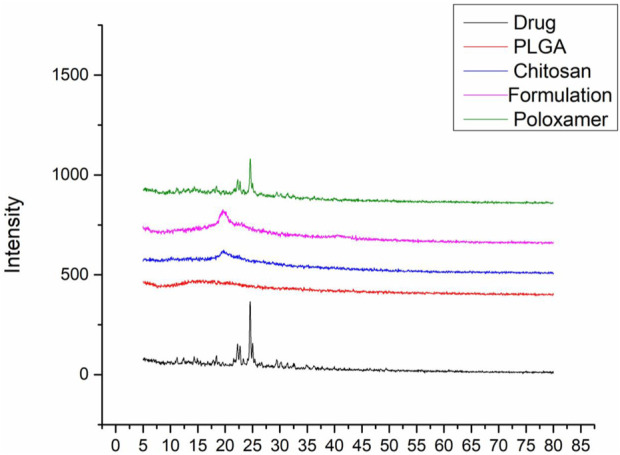
Overlay of the XRD pattern of sorafenib, PLGA, chitosan, nanoformulation, and poloxamer 407.

X-ray diffraction patterns of sorafenib, PLGA, chitosan, poloxamer 407, and nanoformulations were carried out using an X-ray diffractometer (JDX-3532, Jeol, Japan). As shown in Figure 2, the XRD pattern of sorafenib and poloxamer 407 exhibited sharp peaks before the 30° angle, which shows its crystalline nature. Figure 2 illustrates that no peaks were seen for PLGA or chitosan, indicating their amorphous nature. Figure 2 also indicates that the XRD pattern of the sorafenib-loaded nanoformulations has no significant peaks.

### 3.2 Nanoparticle preparation and characterization

**FIGURE 3:**
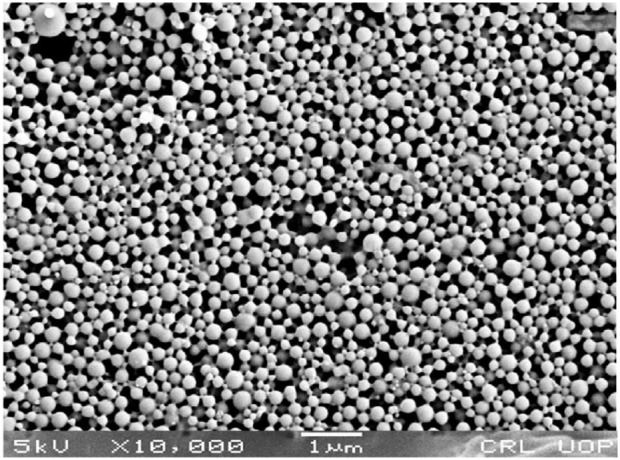
SEM image of the sorafenib-loaded PLGA nanoformulation.

The sorafenib-loaded PLGA–chitosan nanoparticles were prepared with various concentrations of poloxamer, as shown in Table 3. In this study, the particles ranged from 151 to 262 nm for 0.5%, 1%, 1.5%, and 2% poloxamer 407. As illustrated in Table 4, the zeta potential of the nanoparticles was measured to be positive. Additionally, the particle size was in the range of 200 nm, as shown in Table 4; Figure 3. Size, zeta potential, PDI, drug loading, and encapsulation efficiency were analyzed in order to optimize the nanoformulation (Table 4).

**TABLE 3:** Sorafenib formulations with PLGA, chitosan, and poloxamer (0.5, 1, 1.5, and 2%).

S.No.	Code	Sorafenib (mg)	PLGA (mg)	Chitosan (w/v) (2 mL)	Poloxamer 407 in % (13 mL)	Time (min)	Temp. (°C)	Sonication speed (%)
1	Polox38	1	5	0.2	0.5	1	25	99
2	Polox39	2	5	0.2	0.5	1		"
3	Polox40	3	5	0.2	0.5	1		"
4	Polox41	4	5	0.2	0.5	1		"
5	Polox42	1	5	0.2	1	1		"
6	Polox43	2	5	0.2	1	1		"
7	Polox44	3	5	0.2	1	1		"
8	Polox45	4	5	0.2	1	1		"
9	Polox46	1	5	0.2	1.5	1		"
10	Polox47	2	5	0.2	1.5	1		"
11	Polox48	3	5	0.2	1.5	1		"
12	Polox49	4	5	0.2	1.5	1		"
13	Polox50	1	5	0.2	2	1		"
14	Polox51	2	5	0.2	2	1		"
15	Polox52	3	5	0.2	2	1		"
16	Polox53	4	5	0.2	2	1		"

**TABLE 4:** Nanoformulation of sorafenib with PLGA, chitosan, and poloxamer (0.5, 1, 1.5, and 2%).

No.	Drug: PLGA (mg)	Chitosan (% w/v)	Poloxamer 407 (%)	Size (nm)	PDI	ZP (mv)	(%) EE	(%) DL
Polox38	1:5	0.2	0.5	151 ± 11.12	0.279 ± 0.02	5.7 ± 0.01	47	7.4
Polox39	2:5	0.2	0.5	159 ± 10.27	0.129 ± 0.03	5.77 ± 1.2	56	7.7
Polox40	3:5	0.2	0.5	160 ± 8.65	0.163 ± 0.03	6.7 ± 0.34	53	13.7
Polox41	4:5	0.2	0.5	161 ± 14.38	0.119 ± 0.02	6.72 ± 1.4	62	19.1
Polox42	1:5	0.2	1.0	166 ± 21.32	0.148 ± 0.04	7.02 ± 1.8	67	14.6
Polox43	2:5	0.2	1.0	169 ± 7.64	0.193 ± 0.02	16 ± 0.04	69	9.7
Polox44	3:5	0.2	1.0	173 ± 17.66	0.165 ± 0.01	16 ± 1.52	71	10.8
Polox45	4:5	0.2	1.0	177 ± 25.15	0.187 ± 0.03	17 ± 0.22	75	11.2
Polox46	1:5	0.2	1.5	181 ± 11.36	0.192 ± 0.03	19 ± 0.7	81	8.1
Polox47	2:5	0.2	1.5	187 ± 12.74	0.147 ± 0.01	20 ± 1.5	79	7.6
Polox48	3:5	0.2	1.5	189 ± 24.22	0.155 ± 0.02	23 ± 0.56	74	7.9
Polox49	4:5	0.2	1.5	199 ± 18.41	0.202 ± 0.04	30.7 ± 0.08	76	8.2
Polox50	1:5	0.2	2.0	200 ± 13.13	0.233 ± 0.01	32 ± 0.11	87	8.8
Polox51	2:5	0.2	2.0	237 ± 16.61	0.160 ± 0.03	48 ± 0.13	75	9.0
Polox52	3:5	0.2	2.0	244 ± 23.63	0.149 ± 0.04	49 ± 0.11	69	13.4
Polox53	4:5	0.2	2.0	262 ± 14.49	0.079 ± 0.04	66 ± 0.24	64	11.1

### 3.3 Drug loading and encapsulation efficiency

The EE/DL of the nanoparticles (Polox50) was 87% (Table 4).

### 3.4 *In vitro* analysis

#### 3.4.1 Drug release

**FIGURE 4:**
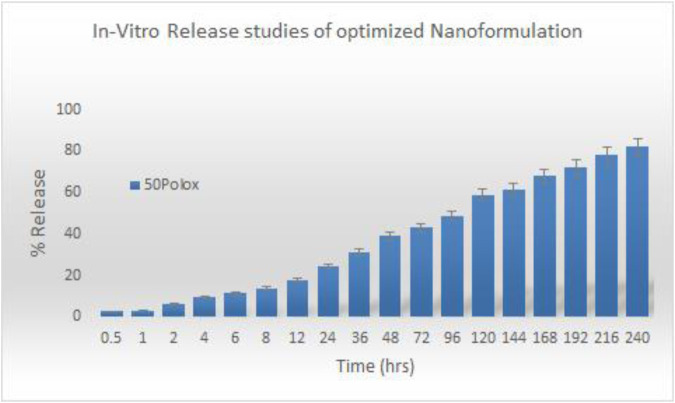
*In vitro* release study of the optimized nanoformulation.

To find the *in vitro* profile of sorafenib nanoformulations, release experiments were conducted utilizing the dialysis diffusion technique. At certain intervals (0.5, 1, 2, 4, 6, 8, 12, 24, 36, 48, 72, 96, 120, 144, 168, 192, 216, and 240 h), samples were taken out, and their drug release was examined. Figure 4 illustrates the bi-phasic release pattern seen in all sorafenib-loaded PLGA nanoformulations. This pattern is marked by an initial burst release within the first 24 h, followed by a constant steady release. After a 24-h period, the sorafenib burst release from nanoformulations was 24.5% ± 0.12% for Polox50. At 240 h, the drug release was 82% ± 0.05% for Polox50.

#### 3.4.2 Kinetics

Several models, including zero-order, first-order, Higuchi, Hixson–Crowell, and Korsemyer–Peppas models, were used to calculate release kinetics. The resulting regression coefficient (R2) values and medication release from the Polox50 nanoformulation that best fit the Higuchi model were found to be those with the highest regression, as shown in Table 5 and Figure 4.

**TABLE 5:** *In vitro* drug release kinetics of the optimized nanoformulation.

Formulation	First-order (R^2^)	Zero-order (R^2^)	Hixson–Crowell (R^2^)	Higuchi (R^2^)	Korsmeyer–Peppas (R^2^)	(n*)
Polox50	0.6714	0.936	0.9636	0.9947	0.9923	0.61

#### 3.4.3 Cytotoxicity studies

**FIGURE 5:**
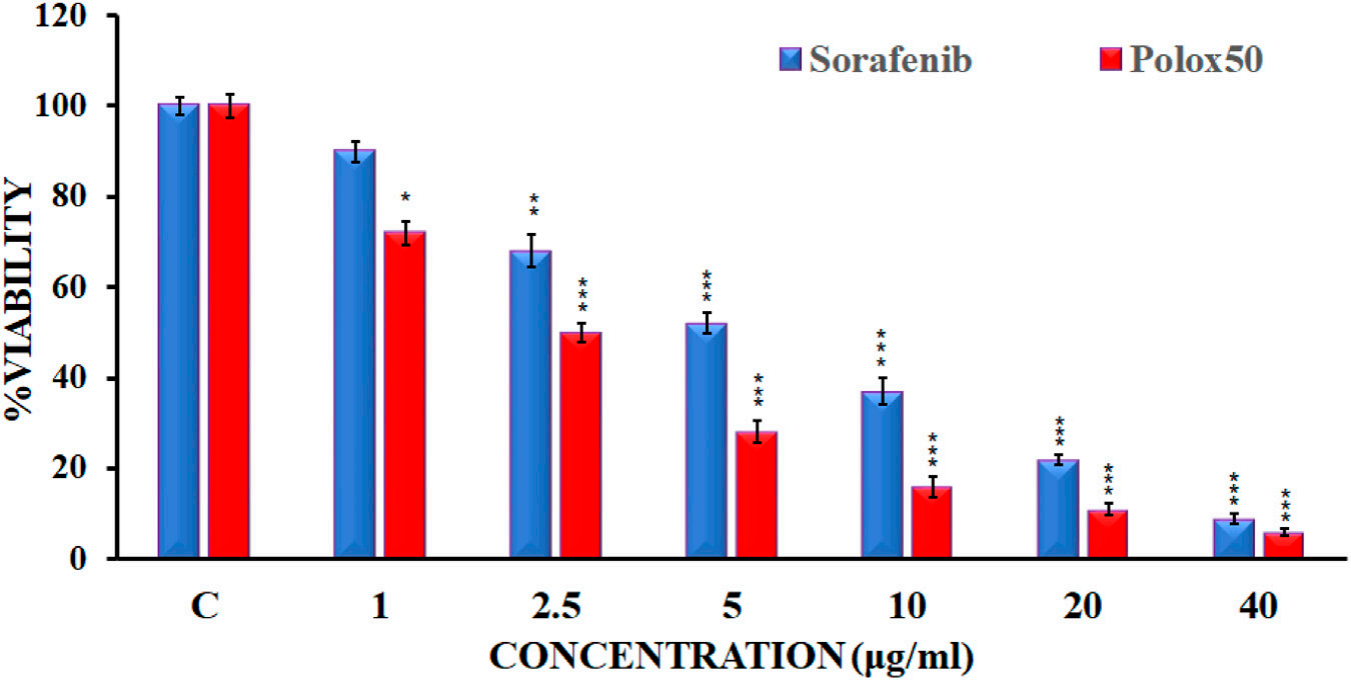
Cell viability of HepG2 cell lines by the reference drug and sorafenib nanoformulation.

An MTT assay was conducted against the HepG2 cell line in order to evaluate sorafenib nanoparticles. In order to determine the suppression of cancer cell proliferation, sorafenib and its nanoparticles were tested against the human liver cancer cell line HepG2 at various concentrations (1 μg/mL, 2.5 μg/mL, 5 μg/mL, 10 μg/mL, 20 μg/mL, and 40 μg/mL). Figure 5 illustrates the MTT experiment used to measure the reduction in cancer cell viability brought on by cytotoxic drugs.

### 3.5 *In vivo* analysis

#### 3.5.1 Pharmacokinetic analysis

**FIGURE 6:**
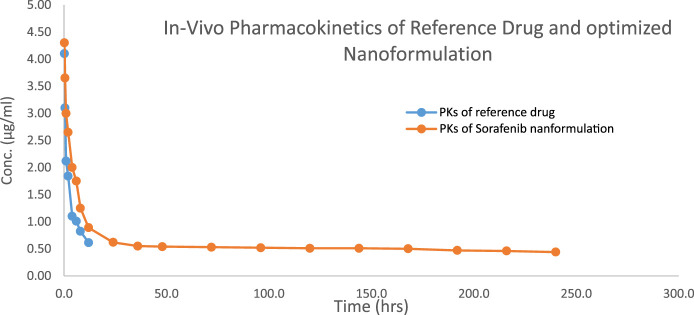
Concentration vs. time profile of the sorafenib nanoformulation with poloxamer 407.

The 1.5–2 kg test rabbits were utilized as an experimental model for the analysis of different *in vivo* pharmacokinetic parameters of the sorafenib nanoformulations. Two injections of chosen sorafenib nanoformulations (2 mg/kg body mass) were administered into the marginal ear vein. The commercially available sorafenib formulation (Nexavar) was used as the reference formulation. The reported RP-HPLC UV method (Khan et al., 2023) was effectively used for the analysis of formulation and reference drug (Nexavar^®^). PK-summit-^®^ non-compartmental analysis was used to assess the results. The results are shown in Table 6; Figure 6.

**TABLE 6:** Pharmacokinetics parameters of sorafenib nanoformulation

Parameter	C_max_	AUC_0-t_	AUMC∞	MRT	t½	Vd	Cl
	μgml^-1^	μghrml^-1^	µghr^2^ml^-1^	Hr	Hr	ml	mlh^-1^ kg^-1^
References/control	4.1 ± 0.011	15.0 ± 0.014	282.3 ± 1.12	12.0 ± 0.05	9.54 ± 0.14	5,881.1 ± 1.56	426.856 ± 0.098
Sorafenib Polox50	4.3 ± 0.048	140.3 ± 1.94	200,923.8 ± 537.15	527.9 ± 1.78	374.09 ± 3.34	14,288.2 ± 0.52	26.469 ± 0.014
*p*-value		0.001***	0.001***	0.001***	0.001***	0.001***	0.001***

## 4 Discussion

The compatibility studies between the APIs and excipients are an important step in the pre-formulation stage of dosage form development. The possible interactions, including both physical and chemical interactions, may affect the stability, structure, and absorption of the formulation, leading to a compromise in the safety and therapeutic response of the drugs (Bharate et al., 2016) The interactions between the different polymers and stabilizers employed in the preparation of nanoformulations with the APIs have the potential to disrupt the stability of the nanoparticles. The physical consistency and drug excipient compatibility analysis were assessed through FTIR studies to prevent these potential interactions. When a medication is stored in harsh conditions, its concentration may decrease as a result of drug deterioration. Humidity and temperature can cause incompatibilities in substances and materials (Xie and Taylor, 2017). Over the course of the first month, the composition of the mixtures did not change in the dosage form (Szymańska and Winnicka, 2015).

The bands in the FTIR spectra were due to the presence of C–Cl, C=O of the carbonyl group, C=O of the amide group, and N–H group attributed to the stretching of the drug. The chitosan FTIR bands at 618, 1,425, and 3,275 cm^−1^ were further noted in the FTIR spectra of sorafenib nanoformulation, representing the C=N, CO–NH_2_ and NH_2_ stretching vibrations in chitosan (Ruman et al., 2021; Mansur et al., 2008; Khan et al., 2023).

The FTIR spectrum of the sorafenib-loaded PLGA–chitosan nanoparticle revealed distinctive peaks for sorafenib, indicating that the drug was fully encapsulated by the polymer. However, the primary peaks for PLGA, chitosan, and poloxamer remained unchanged, suggesting that there was no interaction detected in the mixtures. When visually assessing the specimen, there was neither a specific change observed in the color nor physical consistency, indicating that the active components and medications were congruent with one another. Polox50 was chosen because of its small particle size, strong positive zeta potential, EE of more than 80%, and PDI of less than 0.3. These formulations underwent further assessment for their physicochemical properties. The findings indicate that the combination of poloxamer and the medication caused the size of the nanoparticles to increase. When poloxamer 407’s concentration was increased from 0.5% to 2%, while keeping the concentration of PLGA and chitosan constant, the encapsulation efficiency increased (Table 4) (Sakhi et al., 2022; Khan et al., 2023).

A rise in drug concentration resulted in a corresponding increase in the encapsulation efficiency. Nanoformulations with encapsulation efficiencies of more than 80% were chosen for further studies. The external aspect of the nanoparticles made with poloxamer was ball-shaped, which was also reported by Deshkar et al. (2021). The surface morphology of the selected nanoformulation was spherical, as confirmed by SEM (Figure 3). Based on factors like EE, zeta potential, surface charge, PDI, and particle size, the F1 (Polox50) was chosen for further studies.

The XRD study was conducted to detect the status of sorafenib (crystalline or amorphous) after encapsulation by the polymer. Since there was no distinctive peak in the XRD pattern of the sorafenib-loaded PLGA–chitosan nanoformulations, it can be concluded that sorafenib was entirely contained and fully encapsulated in the polymer (de Oliveira Fortes et al., 2012; Wei et al., 2009).

The findings of *in vitro* studies show that the developed nanoformulations have a bi-phasic release pattern, with an initial burst release during the first 24 h, followed by progressive release. The gradual release of sorafenib from nanoparticles is mostly dependent on drug diffusion and matrix erosion, and it is caused by the gradual breakdown of PLGA and chitosan. According to Singh and Lillard Jr (2009), “The diffusion process is thought to be responsible for the gradual release of the drug, which is confined in the polymeric core of nanoparticles, while poorly adsorbed/entrapped drug on the polymeric matrix outcomes in quick release.” The observation was made that the Higuchi model best matches the drug release from the Polox50 nanoformulation based on the higher regression coefficient (R2 = 0.9947) value. When measured at 60% release concentration, the “n” value primarily demonstrates the mechanism of drug release from the polymeric matrix. The findings indicate that diffusion is the most often used release mechanism, followed by erosion. According to Kamaly et al. (2016), the optimized formulations exhibited non-Fickian diffusion.

The findings of the MTT assay indicated that sorafenib and its nanoparticle Plox50 clearly show commanding activity with an IC_50_ value of 3.9 ± 0.15 μg/mL against the HepG2 cell line. The MTT test shows that compound C50 has more cytotoxicity toward HepG2 cell lines and is more potent. Data from the MTT study revealed that the novel synthetic Polox50 nanoparticles of sorafenib have more potential to treat hepatocellular carcinoma. Therefore, further studies are required to find the exact mechanism of action of this nanoparticle through which it enhances the potency of sorafenib.

Various pharmacokinetic parameters such as Cmax, AUC, AUMC, MRT, t_1/2_, and Vd were greatly increased, whereas Cl was reduced in comparison to the conventional reference formulation of sorafenib (Table 6; Figure 6), which led to enhanced bioavailability, targeting, and efficacy for the treatment of hepatocellular carcinoma.

## 5 Conclusion

In this study, sustained-release sorafenib-loaded polymeric nanoparticles were synthesized utilizing PLGA, poloxamer 407, and chitosan using the solvent evaporation method. The selected nanoformulations were obtained in the required particle size (200 nano-meters), PDI, zeta potential (+32 mv), and encapsulation capability (˃80%). The surface of the nanoparticles was spherical as observed by scanning electron microscopy. The medication was shown to be well-encapsulated within the formulation according to XRD measurements.

The pharmacokinetic parameters of sorafenib-loaded polymeric nanoformulations exhibit enhanced values of MRT, AUC, t_1/2_, and V_d_, while Cl was found to be reduced compared to that of commercially obtainable sorafenib nanoformulation; thus reducing the dose frequency and leading to improved patient compliance. HepG2 cancer cell lines were used to test the cytotoxicity of the chosen formulations. Cell viability significantly decreased with the increasing drug concentration and incubation period. The developed formulations have better bioavailability, targeting, and efficacy for the treatment of hepatocellular carcinoma compared to those of conventional formulations of sorafenib.

## Data Availability

The original contributions presented in the study are included in the article/Supplementary Material; further inquiries can be directed to the corresponding author.
