# Supplementary material for: Formulation development, characterization, and evaluation of sorafenib-loaded PLGA–chitosan nanoparticles
Source: Front Pharmacol. 2024 Oct 9;15:1465363. doi: 10.3389/fphar.2024.1465363 (PMC11496126; doi:10.3389/fphar.2024.1465363)
Supplement: Supplementary file 1 [file DataSheet1.docx]

**Supplementary Data**

**Table Supplementary Data**

| **S.No.** | **Weight of Drug (mg)** | **Weight of NPs** | **Weight of Drug in NPs** | **EE (%)** | **DL (%)** |
| --- | --- | --- | --- | --- | --- |
| 1 | 1 | 6.3 | 0.4 | 47 | 7.4 |
| 2 | 2 | 14.5 | 1.1 | 56 | 7.7 |
| 3 | 3 | 11.6 | 1.5 | 53 | 13.7 |
| 4 | 4 | 12.9 | 2.4 | 62 | 19.1 |
| 5 | 1 | 4.5 | 0.6 | 67 | 14.6 |
| 6 | 2 | 14.2 | 1.3 | 69 | 9.7 |
| 7 | 3 | 19.7 | 2.1 | 71 | 10.8 |
| 8 | 4 | 26.7 | 3.1 | 75 | 11.2 |
| 9 | 1 | 10.1 | 0.8 | 81 | 8.1 |
| 10 | 2 | 20.7 | 1.5 | 79 | 7.6 |
| 11 | 3 | 28.1 | 2.2 | 74 | 7.9 |
| 12 | 4 | 37.0 | 3.0 | 76 | 8.2 |
| 13 | 1 | 9.8 | 0.8 | 87 | 8.8 |
| 14 | 2 | 16.6 | 1.5 | 75 | 9.0 |
| 15 | 3 | 15.4 | 2.0 | 69 | 13.4 |
| 16 | 4 | 23.0 | 2.5 | 64 | 11.1 |

**SEM Images**

**
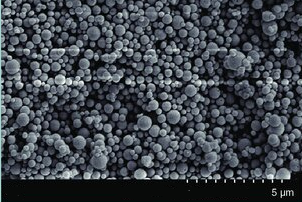
**

**
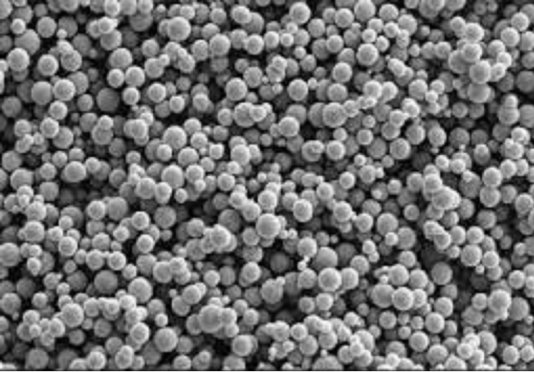
**

**
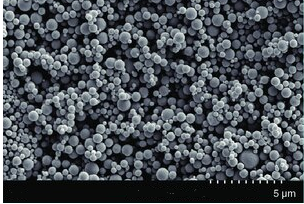
**

**Data for Figure 4**

| Time (hr) | Polox50 | |
| --- | --- | --- |
|  | **% Release** | **SD** |
| 0.5 | 2.8 | 0.29 |
| 1 | 3.2 | 0.04 |
| 2 | 6.2 | 0.35 |
| 4 | 9.6 | 0.84 |
| 6 | 11.4 | 0.78 |
| 8 | 14 | 0.90 |
| 12 | 18 | 0.53 |
| 24 | 24.5 | 0.77 |
| 36 | 31 | 0.30 |
| 48 | 38.9 | 0.65 |
| 72 | 43 | 0.03 |
| 96 | 48.6 | 0.01 |
| 120 | 59 | 0.39 |
| 144 | 61.3 | 0.21 |
| 168 | 67.9 | 0.16 |
| 192 | 72 | 0.42 |
| 216 | 78 | 0.33 |
| 240 | 82 | 0.34 |
